# 3,3′-Diaminobenzidine staining interferes with PCR-based DNA analysis

**DOI:** 10.1038/s41598-018-19745-9

**Published:** 2018-01-19

**Authors:** Christian Dölle, Laurence A. Bindoff, Charalampos Tzoulis

**Affiliations:** 1 Department of Neurology, Haukeland University Hospital, Bergen, Norway; 2 Department of Clinical Medicine, University of Bergen, Bergen, Norway

## Abstract

3,3′-Diaminobenzidine (DAB) is a widely used chromogen in histological staining methods and stained tissue is often used in downstream molecular analyses such as quantitative PCR (qPCR). Using microdissected muscle fibers from sequential muscle sections stained by DAB-dependent and -independent methods, we show that DAB exerts a strong inhibitory effect on qPCR-based mitochondrial DNA quantification. This effect introduces a significant bias in the estimation of mitochondrial DNA copy number and deletion levels between DAB-positive and -negative fibers. We reproduce our findings in microdissected neurons from human brain tissue, suggesting a general effect of DAB staining on PCR analyses independent of the underlying tissue or cell type. Using an exogenous DNA template added to tissue samples we provide evidence that DAB-staining predominantly interferes with the tissue-derived DNA template rather than inhibiting DNA polymerase activity. Our results suggest that DAB-based staining is incompatible with PCR-based quantification methods and some of the previously reported results employing this approach should be reconsidered.

## Introduction

The chromogen 3,3′-Diaminobenzidine (DAB) is commonly employed as a staining agent in histochemical (HC) and immunohistochemical (IHC) procedures performed for clinical and research purposes. To act as a dye, DAB is oxidised and converted to an insoluble polymer, which precipitates as a dark brown pigment at the reaction site allowing visualization of the target molecule^1,2^. In immunohistochemistry, DAB serves as substrate for a peroxidase enzyme coupled to a primary or secondary antibody. Histochemical staining methods using DAB include peroxisomal staining exploiting the presence of catalase in these organelles^3^, and mitochondrial cytochrome c oxidase (COX, also known as respiratory complex IV) activity staining^1,2^.

COX histochemistry is one of the standard methods for assessing mitochondrial function in tissue, either alone or in combination with histochemistry for succinate dehydrogenase (SDH). In the COX reaction, DAB is oxidised and functions as electron donor for cytochrome c, which in turn transfers its electrons to respiratory complex IV. In the SDH reaction, succinate is oxidised, and transfers its electrons via succinate dehydrogenase (respiratory complex II) in the presence of phenanzine methosulphate (PMS) to the final electron acceptor nitroblue tetrazolium (NBT), which forms a blue precipitate^1^. While three subunits of COX are encoded by mitochondrial DNA (mtDNA), SDH is entirely nuclear encoded. The identification of COX negative and positive cells using double COX/SDH histochemistry is particularly useful for assessing mitochondrial respiratory dysfunction due to mtDNA damage, which can be primary, i.e. an mtDNA mutation, or secondary, e.g, multiple mtDNA deletions caused by a nuclear gene defect^1,4,5^.

Combined COX/SDH histochemistry is now the standard method for identifying cells with high levels of heteroplasmic mtDNA mutations. Indeed, establishing segregation of mutation with the histochemical defect (i.e., COX deficiency) is used as one of the criteria for pathogenicity. It is common, therefore, to perform quantitative and qualitative mtDNA analyses in individual microdissected cells or areas of tissue that have been stained using this technique. COX positive and negative cells or regions are microdissected, lysed and assessed for mtDNA deletions and copy number by quantitative PCR (qPCR)^6^.

qPCR analyses are established standard methods to detect and quantify specific DNA fragments or sequences, and are also widely applied to mtDNA analysis^7^. While the PCR technique is rapid, specific and highly sensitive, it is also prone to inhibitors that may affect sensitivity or lead to false-negative results^8,9^. PCR-inhibitors include a diverse spectrum of substances (e.g., organic compounds, metal ions, detergents and biological sample debris) and act in different ways, for example by altering the DNA template, inhibiting the polymerase activity or affecting fluorophores used for signal detection^9^. It is therefore important to evaluate putative confounding factors and process DNA samples to minimize inhibition.

A potential confounder in experiments combining qPCR with histochemical staining is the large difference in DAB pigment present in COX-positive cells versus COX-negative cells. Thus, if DAB, or its oxidation product, affects any of the steps between dissection and qPCR amplification, this could introduce a substantial bias. Indeed, in our experience, qPCR on DNA extracted from DAB-stained material tends to behave differently, suggesting a lower reaction efficiency. Moreover, concern regarding interference by DAB staining with quantitative DNA assessment has been raised previously^10^. Other studies, however, have reported no untoward effect of DAB staining on qPCR analysis^11^. To our knowledge, no systematic analysis of the influence of DAB staining on PCR-based methods has been carried out, and given the widespread use of DAB-stained material, we believed this required urgent clarification. We therefore examined how DAB stained tissue samples perform in DNA analysis and show that DAB staining negatively influences quantitative PCR analysis of tissue DNA samples.

## Results

**Figure 1:**
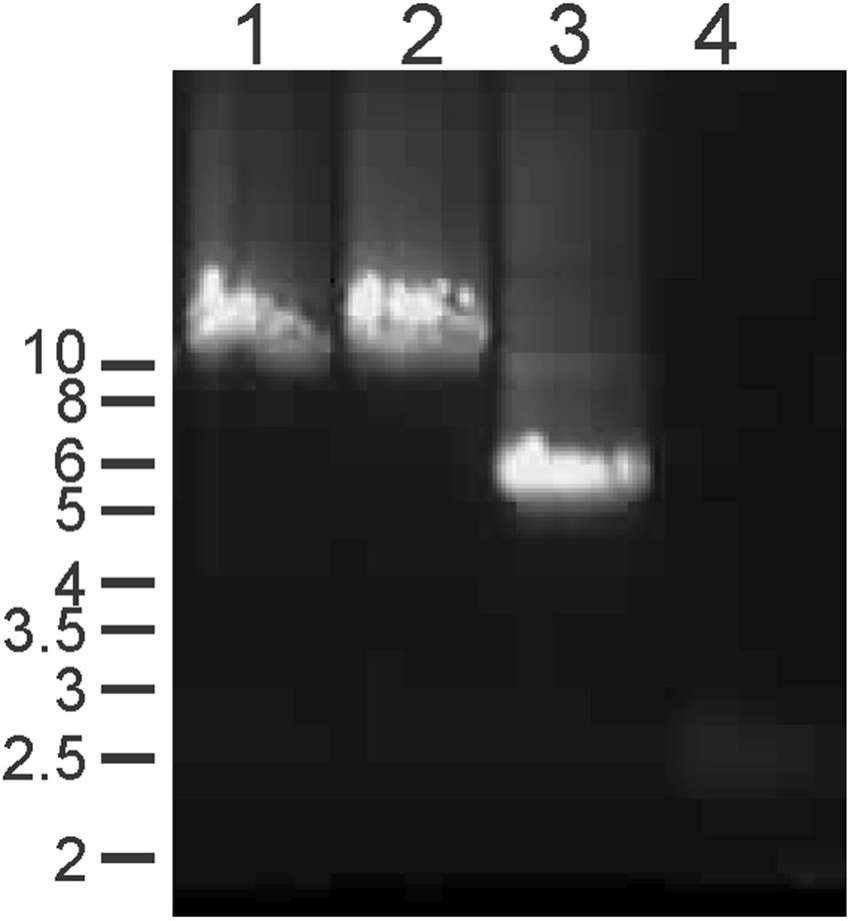
Detection of a single mtDNA deletion in muscle biopsy. DNA extracted from muscle biopsy was analysed for mtDNA deletion by long range pcr and amplicons resolved on an agarose gel (0.7%). The image shows the pcr product amplified from control (1 and 2) and patient (3) tissue, as well as a non-template control (NC). The patient sample shows a ~5.5 kb single mtDNA deletion.

In order to investigate the putative effect of DAB staining on qPCR, we performed quantitative and qualitative mtDNA analyses in individual fibers from a skeletal muscle biopsy of a patient with mitochondrial myopathy due to single mtDNA deletion. The patient biopsy showed ~30% COX negative muscle fibers. Long-range PCR and qPCR of mtDNA from homogenised muscle confirmed the presence of a 5.5 kb mtDNA deletion (Fig. 1) affecting ~67% of the total mtDNA population (not shown).

**Figure 2:**
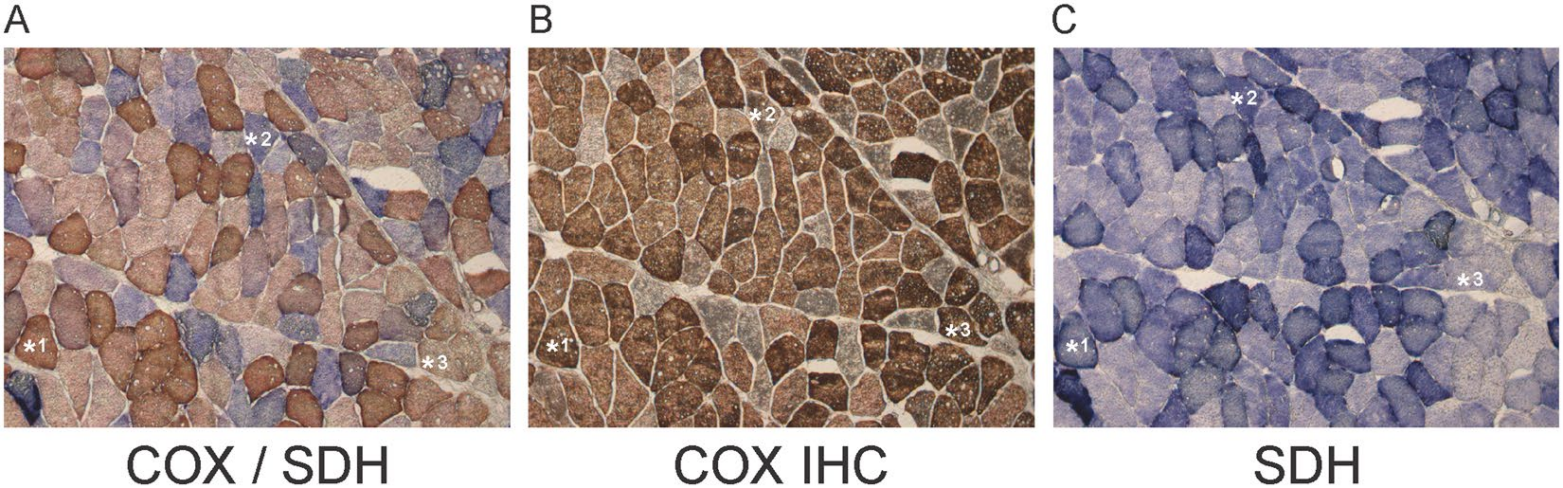
Histochemical and immunohistochemical assessment of mitochondrial respiratory function in muscle fibers. Consecutive sections (10 µm) of the same muscle biopsy were stained either by COX/SDH histochemistry (**A**), SDH histochemistry (**C**) or COX immunohistochemistry (**B**). Asterisks indicate identical muscle fibers in the different sections for orientation. Samples were taken from strongly stained COX-positive (e.g., *1) and clearly COX-negative fibers (e.g., *2) by laser micro dissection and analysed by qPCR. Lightly stained COX-positive fibers (e.g., *3) were not included.

Serial muscle sections were prepared and stained by DAB-free (SDH histochemistry alone) or DAB-dependent (COX/SDH histochemistry and COX immunohistochemistry) methods (Fig. 2). As the amount of DAB pigment in a fiber varies depending on the levels of COX protein, we distinguished between COX positive and COX negative fibers and analysed these separately. Using serial sections, we were able to identify and sample the same muscle fiber stained by the three different techniques. Each fiber was then sampled by laser microdissection from each of the three serial sections and analysed by qPCR for mtDNA copy number and deletion levels.

**Figure 3:**
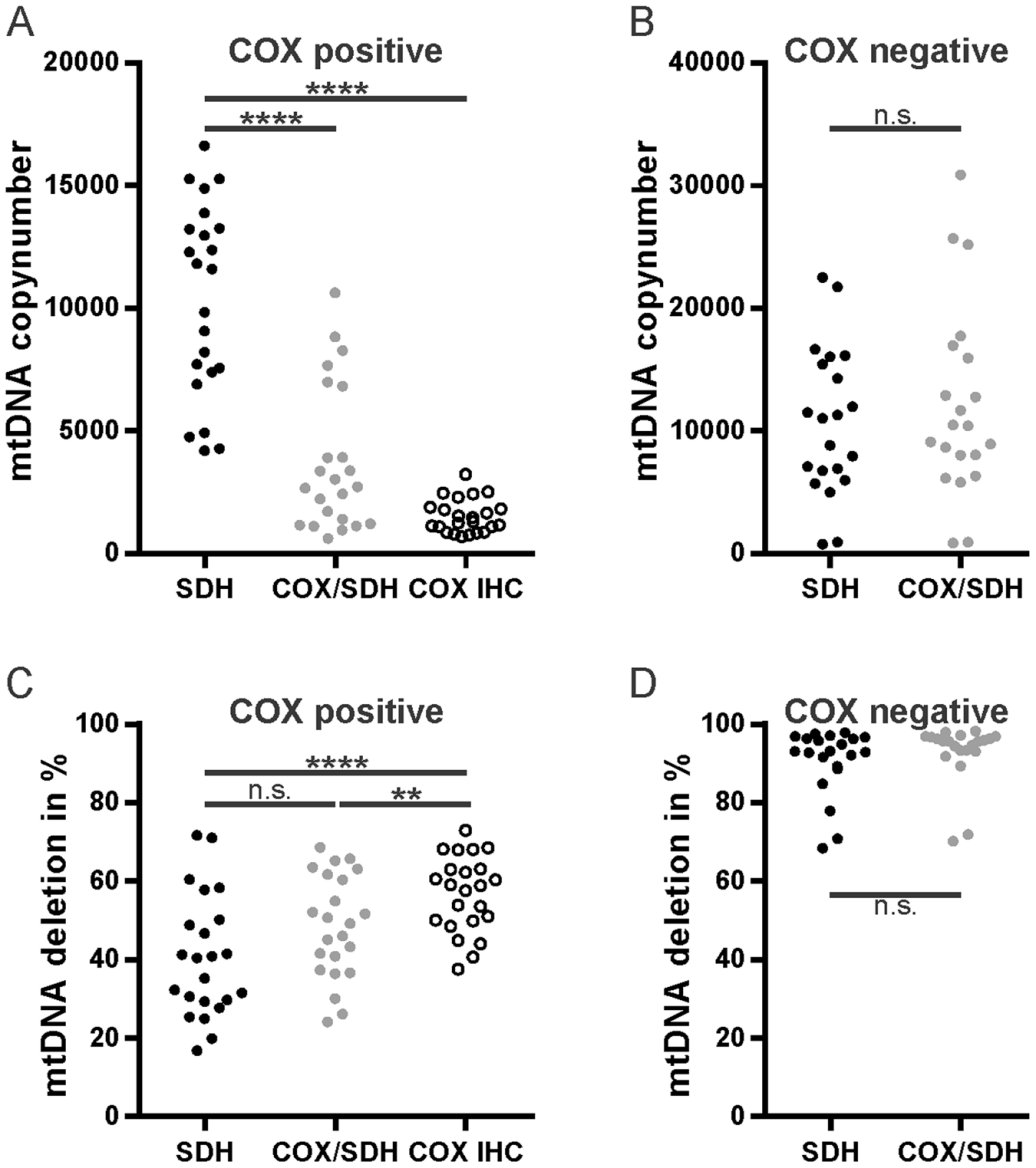
DAB staining interferes with PCR-based quantitative DNA analysis. Samples from COX-positive (n = 23) and COX negative (n = 21) muscle fibers were analysed for their mtDNA content and deletion level. COX positive fibers were analysed following three different staining methods (SDH single histochemical activity stain (SDH), COX/SDH dual histochemical activity stain (COX/SDH) and COX-immunohistochemistry (COX IHC)). COX negative fibers were analysed following SDH single- and COX/SDH-dual histochemical activity staining. (**A**,**B**) Absolute mtDNA copy number analysis following the indicated staining procedure in COX positive (**A**) and COX negative (**B**) muscle fibers. ****< 0.0001; n.s.: not significant. (**C**,**D**) MtDNA deletion analysis in COX positive and COX negative fibers upon different stainings. ****< 0.0001; **< 0.01; n.s.: not significant.

We then compared the different staining methods within each individual fiber. Importantly, COX positive fibers exhibited a significantly higher mtDNA copy number when stained for SDH alone, compared to the DAB-containing COX/SDH histochemistry or IHC (paired ANOVA: SDH vs COX/SDH P = 6.35 * 10^−9^; SDH vs IHC P = 8.60 * 10^−11^; COX/SDH vs IHC P = 0.002; Fig. 3A). Conversely, COX negative fibers showed no significant difference in mtDNA copy number between SDH (10690 ± 6004) and COX/SDH (12066 ± 7775) staining (t-test: *P* = 0.62; Fig. 3B).

The staining method had a less pronounced, but still significant impact on measured levels of mtDNA deletion. COX positive fibers showed overall lower mtDNA deletion levels in SDH stained sections compared to COX/SDH stained sections or IHC (paired ANOVA: SDH vs COX/SDH P = 0.038; SDH vs IHC P = 6 * 10^−6^; COX/SDH vs IHC P = 0.006; Fig. 3C). COX negative fibers exhibited similar levels of mtDNA deletion independently of the staining method (SDH: 90.7 ± 8.5%, COX/SDH: 92.9 ± 7.6%; t-test *P* = 0.195; Fig. 3D).

**Figure 4:**
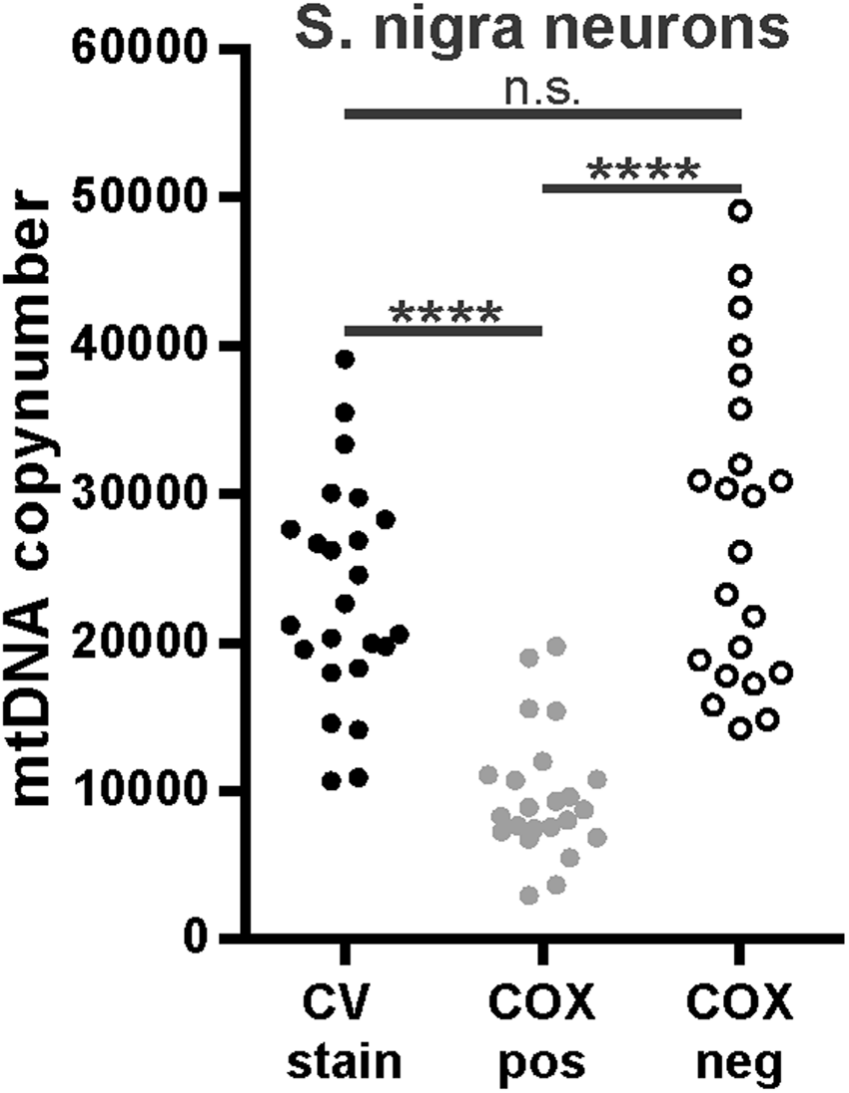
DAB staining affects PCR-based quantitative DNA analysis in neurons. COX-positive (n = 23) and COX negative (n = 22) substantia nigra neurons from three individuals were analysed for their mtDNA content and compared with Cresyl violet stained neurons (n = 24) from the same individuals (values taken from^15^). ****< 0.0001; n.s.: not significant.

To establish whether the observed DAB effects were influenced by the underlying cell type, we replicated our experiment in brain tissue from patients with Parkinson disease. We assessed mtDNA in microdissected neurons from human substantia nigra sections, which had been stained with COX/SDH histochemistry or cresyl violet. Similar to muscle, neuronal mtDNA copy number was significantly higher (t-test *P* < 0.0001) in cresyl violet stained (23256 ± 7374) and COX negative (27796 ± 10551) neurons compared to COX positive neurons (9658 ± 4297). There was no significant difference between cresyl violet and COX negative stained neurons (t-test *P* = 0.208; Fig. 4).

**Figure 5:**
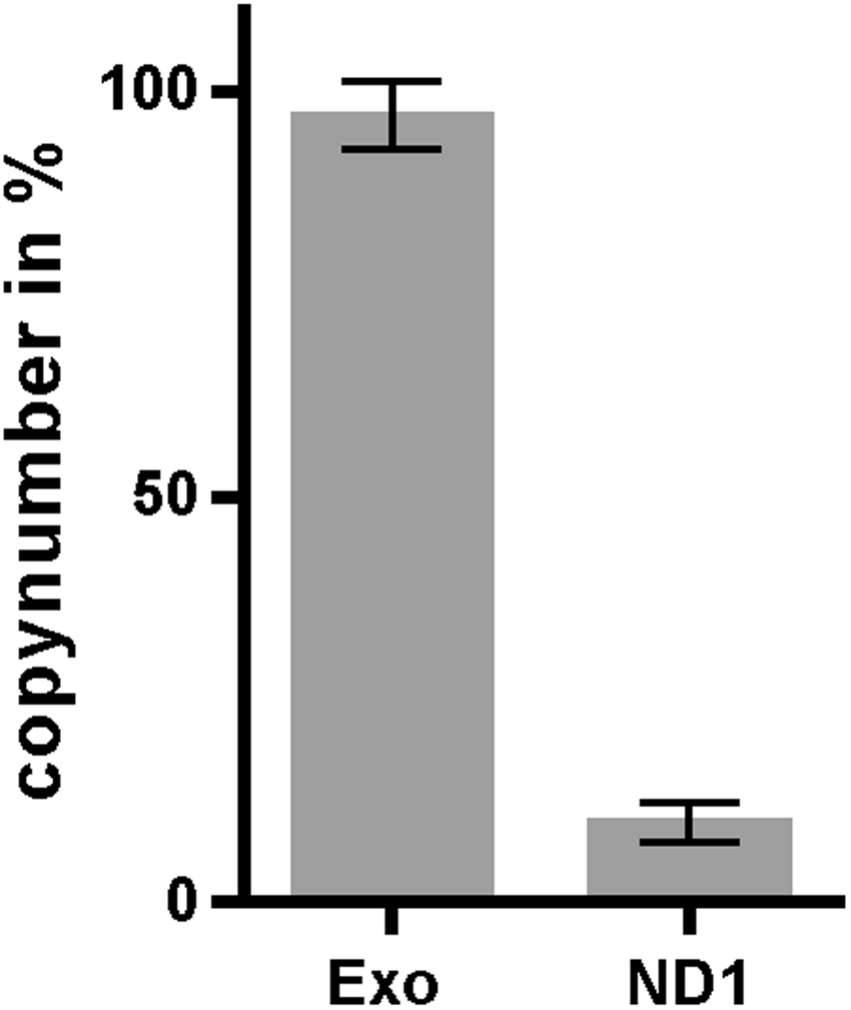
DAB staining affects quantitative analysis of endogenous, but not exogenous DNA. Samples from COX positive fibers (n = 10), stained either by dual histochemical activity stain (COX/SDH) or single histochemical activity stain (SDH), were spiked with a predetermined amount of exogenous DNA (Exo) and subjected to qPCR analysis. The graph shows target gene copy number of the COX/SDH stained samples in percent relative to the SDH stained sample (set to 100%). In COX/SDH sections, detection of endogenous mtDNA target ND1 (ND1) was strongly reduced (9.95% of SDH stained sample), whereas detection of exogenous control DNA (Exo) was nearly unaffected (97% of SDH stained sample). Bars show mean ± SD.

In order to gain insight into the putative mechanism of the observed inhibition, we analysed samples derived from COX-positive muscle fibers which had been stained with DAB-free (SDH histochemistry alone) or DAB-dependent (COX/SDH histochemistry) methods and spiked with a predetermined amount of an exogenous DNA sample. Consistent with our previous experiments, the endogenous, tissue-derived DNA target (ND1) showed an approximately 90% lower amplification signal in DAB-stained sections compared with DAB-free sections of the same muscle fibers (Fig. 5). In contrast, the exogenous DNA sample was amplified to a similar extent (97% similarity) in DAB-containing and DAB-free samples (Fig. 5). These results suggest that the observed reduction in qPCR signal is due to a direct effect on the DNA template derived from DAB-stained tissue, rather than inhibition of the polymerase or signal detection.

## Discussion

We show that histochemical and immunohistochemical DAB-based staining methods have a profound impact on downstream qPCR applications, resulting in a significant underestimation of the template quantity. Our findings are corroborated by previous observations reporting a similar interference of COX/SDH staining with downstream qPCR quantification of mtDNA in muscle^10^.

We believe that the presence of DAB chromogen is the cause of the qPCR variation, since it is the only common difference between SDH histochemistry and either COX/SDH histochemistry or complex IV immunohistochemistry. This conclusion is supported by our finding that mtDNA measurements from COX negative fibers, which contain little or no DAB, were unaffected. Further, we show that the presence of DAB affects qPCR quantification in a similar fashion in two different tissues, suggesting that this effect is independent of the underlying cell type and therefore likely to apply in all tissues and PCR based quantification applications. Finally, we provide evidence suggesting that DAB interacts with the DNA template rather than generally inhibiting polymerase activity in the PCR reaction.

Incubation of the tissue with a DAB substrate solution alone is not sufficient for interference as that would have affected both COX positive and negative fibers in the COX/SDH stained samples. The fact that only DAB-stained (i.e., COX positive) fibers are affected suggests that it is rather the oxidised and polymerised reaction product of DAB^1,2^ which exhibits the inhibitory effect on the qPCR analysis. This is reflected by a stronger effect in IHC samples, where muscle fibers were more intensely stained.

The mechanism by which DAB affects the amplification of DNA remains unclear; the DAB reaction product may interact with the DNA template, inhibit the enzyme of the PCR reaction, or interfere with the emission and/or registration of the fluorescent signal. Our results suggest a direct interaction of DAB-product with only the tissue-derived DNA, making it less available as PCR template. In muscle samples that were spiked with an exogenous DNA template after tissue staining and lysis, amplification consistently detected the exogenous DNA to a similar extent in both DAB-stained and DAB free lysates. In contrast, amplification of the endogenous, tissue-derived DNA sample was strongly affected by the staining method. We cannot rule out potential additional effects of DAB on the polymerase activity or interference with the fluorescent signal; a slight, but consistent 3% reduction in amplification signal was also observed for the exogenous DNA template. This difference was, however, minor compared to amplification reduction in the endogenous tissue DNA.

Our experiments showed that the presence of DAB chromogen affected mtDNA copy number measurements more than mtDNA deletion assessment. This is due to the fact that mtDNA copy number was determined by comparing samples with or without DAB chromogen to a standard curve that is DAB-free. Thus, the staining status of the tissue confounded the outcome of the analysis. In contrast, mtDNA deletion assessment uses an internal ratio of two different reactions (i.e., ND1 and ND4 amplification) both of which occur within the same sample and are, therefore, exposed to the same concentration of DAB. Nevertheless, there was a significant difference in the measured deletion ratio between DAB-stained and DAB-free samples. Notably, this difference was most pronounced in the case of intensely IHC-stained fibers. This suggests that the DAB effect is not uniform for each reaction and may vary depending on target gene and PCR primer sequence, choice of fluorophore and the amount of DAB present in the reaction. This is an important and novel observation, suggesting that a heterogeneous DAB staining pattern confounds the assessment of not only mtDNA quantity, but also mtDNA deletion levels, due to overestimation of the latter in DAB-stained cells.

In conclusion, DAB-based staining introduces a substantial experimental bias in qPCR-based applications. This is most pronounced when cell populations of different staining profiles are compared, such as COX positive and negative muscle fibers or neurons.

Previous studies employing qPCR for mtDNA assessment in individual muscle fibers have reported that COX negative muscle fibers of patients with single mtDNA deletions, point mutations^12,13^ or aging individuals^14^ contain substantially higher levels of deleted and total mtDNA compared to COX positive fibers. It has been speculated that this may be a compensatory reaction to higher levels of mutation in the COX negative fibers. The upregulation of mtDNA in response to qualitative defects (e.g. point mutations or deletions) is indeed an established phenomenon, confirmed by multiple DAB-independent methods^15,16^. The reported difference between COX positive and negative fibers may, however, have been overestimated due to the confounding effect of DAB-based staining methods on the qPCR.

Our findings suggest that total mtDNA copy number does not differ dramatically between COX positive and -negative fibers. In contrast, the level of mtDNA deletion does. Thus, the amount of wildtype (i.e., non-deleted) mtDNA molecules is the decisive factor for maintaining respiratory function.

Moreover, our results suggest that the difference in mtDNA deletion levels between COX positive and negative fibers may have been underestimated previously, due to an apparent higher measurement in DAB-stained COX positive fibers. Our data suggest the possibility that the previously suggested pathological threshold for an mtDNA deletion ratio of 60%^17,18^ may in fact be even lower, because of inaccurate measurement of the levels of mtDNA deletion in COX positive, DAB-stained fibers. Indeed, we believe that the available evidence is sufficiently robust to require a reassessment of previous assumptions based on studies employing DAB-based staining methods.

It is possible to avoid DAB based staining methods upstream of qPCR-based DNA analyses, at least in some cases. For analyses in muscle tissue, these problems can be circumvented by analyzing sequential sections as employed by us and others^10^. One caveat is the segmental distribution of COX deficiency along individual muscle fibers^10^, but given that segments generally tend to be substantially longer than the typical thickness of a histological section, this limitation should have a negligible impact as long as several fibers are sampled.

Unfortunately, the same solution cannot be applied to smaller cells such as neurons. The use of fluorescently labelled antibodies could circumvent this problem and allow assessment of protein quantity rather than enzymatic activity. However, this too would need optimization to avoid interference with subsequent fluorescence-based qPCR analysis.

## Material and Methods

### Patient material

Muscle from a 50 year old male individual with mitochondrial myopathy due to single mtDNA deletion was used. His clinical features comprised bilateral blepharoptosis, progressive external ophthalmoplegia and a moderate proximal limb myopathy and weakness. The muscle biopsy was conducted on a clinical indication for diagnostic purposes. Single neurons derived from frozen substantia nigra tissue was obtained from three individuals with Parkinson disease from a prospective population-based cohort previously described in detail^19^. Ethical approval by the Regional Ethical Committee of Western Norway and patient informed consent for the use of the biopsy material and brain tissue (REK 2011/1504, REK 131/04 and REK 2010/23) were obtained and experiments were performed in accordance with relevant guidelines and regulations.

### Histochemistry and Immunohistochemistry

10 μm thick, consecutive sections from frozen (−80 °C) blocks of a skeletal muscle biopsy and 20 µm thick sections from frozen blocks of substantia nigra were cut using a cryostat (Leica CM 1950) and mounted on membrane slides 1.0 PEN (Zeiss). Tissue sections were air-dried for 1 hour, and subsequently stained with one of the following staining techniques.

For cytochrome c oxidase/succinate dehydrogenase (COX/SDH) histochemical staining, sections were incubated with ~50 µl of COX-staining solution (0.1 M NaPO_4_, 4 mM 3,3-diaminobenzidine tetrahydrochloride (DAB), 100 µM cytochrome c, 2 mg/ml catalase) for 45 min at 37 °C. After washing 3 times for 5 min in PBS, sections were incubated with ~50 µl SDH staining solution (0.1 M NaPO_4_, 1.5 mM nitroblue tetrazolium (NBT), 130 mM sodium succinate, 0.2 mM phenazine methosulphate, 1 mM sodium azide), for 40 min at 37 °C. The sections were washed 3 times with PBS for 5 min and dehydrated with graded ethanol (75% and 95% for 1 min each and 100% ethanol for 10 min). For SDH staining alone, the same procedure as for COX/SDH staining was carried out, omitting the first steps for COX staining. After staining, the samples were dried completely before sampling.

For immunohistochemical detection of COX, sections were fixed in PBS/4% paraformaldehyde for 30 min at 4 °C. After rinsing with water, endogenous peroxidase activity was blocked by incubation in PBS/3% H_2_O_2_ for 5 min. After washing twice in TBS and once in TBS-T (TBS containing 0.1% Tween 20) for 5 min, sections were blocked with TBS-T/4% BSA for 10 min. Then, sections were incubated with primary antibody (mouse anti MTCO, ab14705 (Abcam)) for 1 hour at RT in TBS-T/4% BSA. Development of IHC was carried out using MACH4 DAB detection kit (Biocare medical) according to the manufacturer’s recommendations. Sections were washed in tap water and dehydrated in graded ethanol solutions (10 sec in 70%, 10 sec in 90% and 5 min in 100% ethanol). Sections were allowed to dry completely before sampling.

### Laser microdissection (LMD) and sample lysis

Laser microdissection was performed on a PALM Laser microdissection microscope (Zeiss). In excess of 40 muscle fibers were individually sampled from all differently stained sections using a preset surface area size. Collected samples were individually lysed in 15 μl lysis buffer (50 mM Tris pH 7.4, 0.5% Tween-20, 200 μg/ml proteinase K) overnight at 56 °C, centrifuged (5 min, 10,000 rpm, 4 °C), incubated for 10 min at 95 °C to inactivate proteinase K, and centrifuged again. Each cell lysate was subsequently used in downstream mtDNA analyses comprising determination of total copy number and fraction of molecules harboring major arc deletions.

### MtDNA copy number and deletion analysis

Total mtDNA copy number and the fraction of major arc deletion were determined in individual, identical muscle fibers from consecutive sections subjected to different staining methods. Copy number and deletion analysis were performed simultaneously in the same reaction, using a duplex qPCR assay to detect a commonly deleted (*MTND4*) and rarely deleted (*MTND1)* targets on the mitochondrial genome as previously described^15^. The following primers, probes and conditions were used. MTND1 forward primer: 5′-CCCTAAAACCCGCCACATCT-3′, MTND1 reverse primer: 5′-GAGCGATGGTGAGAGCTAAGGT-3′, MTND1 hydrolysis MGB probe: 5′-FAM-CCATCACCCTCTACATCACCGCCC-3′. MTND4 forward primer: 5′-CCATTCTCCTCCTATCCCTCAAC-3′, MTND4 reverse primer: 5′-CACAATCTGATGTTTTGGTTAAACTATATTT-3′, MTND4 hydrolysis MGB probe: 5′-VIC-CCGACATCATTACCGGGTTTTCCTCTTG-3′. 2 µl cell lysate were used per qPCR reaction and all samples were run in triplicate. Amplification was performed on a 7500 fast sequence detection system (Life Sciences) using TaqMan Fast Advanced Master Mix containing AmpliTaq® Fast DNA Polymerase (Thermofisher) according to the manufacturer’s instructions. Thermal cycling consisted of one cycle at 95 °C for 20 sec and 40 cycles at 95 °C for 3 sec and 60 °C for 30 sec. For absolute quantification, target amplification was compared to a standard curve made from a serial dilution containing equal amounts of PCR-generated and purified full-length *MTND1* and *MTND4* template (10^2^–10^6^ copies/μl). *MTND1* is rarely deleted and reflects total mtDNA copy number whereas *MTND4* only amplifies molecules not harboring major arc deletions. The percentage of deletion was calculated from the difference of the two as previously described^7^. Blood genomic DNA was used as an internal control in each experiment. Long range PCR for detection of single mtDNA deletion was performed as previously described^20^.

The exogenous DNA template was generated by PCR amplification of a 500 bp fragment of the genomic sequence of the nuclear encoded *APP* gene using primer pairs 5′-TGCTCAACCAGTAAGTATATAATG-3′ and 5′-TGCTCCTATAGGGTCAGTGC-3′ spanning an intron-exon boarder. For qPCR analysis, the following primers and probe were used in a duplex qPCR assay together with *MTND1* primers and probe (see above): APP forward primer: 5′-TGTGTGCTCTCCCAGGTCTA-3′; APP reverse primer: 5′-CAGTTCTGGATGGTCACTGG-3′; APP hydrolysis-MGB probe: 5′-VIC-CCCTGAACTGCAGATCACCAATGTGGTAG-3′. Absolute quantification was achieved as described above using a serial dilution containing equal amounts of PCR-generated and purified APP template and full-length *MTND1* (10^2^–10^6^copies/μl).

### Data availability

All data generated or analysed during this study are included in this article.
